# High-Speed All-Optical Encoder and Comparator at 120 Gb/s Using a Carrier Reservoir Semiconductor Optical Amplifier

**DOI:** 10.3390/nano15090647

**Published:** 2025-04-24

**Authors:** Amer Kotb, Kyriakos E. Zoiros

**Affiliations:** 1 School of Chips, XJTLU Entrepreneur College (Taicang), Xi’an Jiaotong-Liverpool University, Taicang, Suzhou 215400, China; 2 Department of Physics, Faculty of Science, University of Fayoum, Fayoum 63514, Egypt; 3 Lightwave Communications Research Group, Department of Electrical and Computer Engineering, School of Engineering, Democritus University of Thrace, 67100 Xanthi, Greece

**Keywords:** optical encoder and comparator, carrier reservoir semiconductor optical amplifier, Mach–Zehnder interferometer, quality factor

## Abstract

All-optical encoders and comparators are essential components for high-speed optical computing, enabling ultra-fast data processing with minimal latency and low power consumption. This paper presents a numerical analysis of an all-optical encoder and comparator architecture operating at 120 Gb/s, based on carrier reservoir semiconductor optical amplifier-assisted Mach–Zehnder interferometers (CR-SOA-MZIs). Building upon our previous work on all-optical arithmetic circuits, this study extends the application of CR-SOA-MZI structures to implement five key logic operations between two input signals (A and B): $\bar{A}B$, $A\bar{B}$, AB (AND), $\bar{A}\;\bar{B}$ (NOR), and AB + $\bar{A}\;\bar{B}$ (XNOR). The performance of these logic gates is evaluated using the quality factor (QF), yielding values of 17.56, 17.04, 19.05, 10.95, and 8.33, respectively. We investigate the impact of critical design parameters on the accuracy and stability of the logic outputs, confirming the feasibility of high-speed operation with robust signal integrity. These results support the viability of CR-SOA-MZI-based configurations for future all-optical logic circuits, offering promising potential for advanced optical computing and next-generation photonic information processing systems.

## 1. Introduction

The growing demand for ultra-fast and energy-efficient data processing in modern optical communication systems has accelerated the development of all-optical logic gates (AOLGs). These gates eliminate the need for optical-to-electrical conversion, significantly reducing latency and power consumption while improving system throughput, making them indispensable in photonic networks and next-generation computing systems. AOLGs are essential for high-speed data routing and switching and are also increasingly applied in areas such as encryption, neuromorphic computing, and real-time signal processing [1,2,3,4,5,6,7,8,9,10]. While traditional electronic systems have limitations in bandwidth and speed, photonic approaches offer parallelism and high data rates. Semiconductor optical amplifiers (SOAs) have been widely investigated among various technologies due to their nonlinear behavior, compactness, and integrability. However, conventional SOAs suffer from limitations such as slow carrier recovery times and gain saturation effects, which degrade their performance at high bit rates [11]. To overcome these drawbacks, the carrier reservoir semiconductor optical amplifier (CR-SOA) has emerged as a promising alternative. By incorporating an additional reservoir section, the CR-SOA facilitates faster carrier replenishment, leading to improved gain recovery and stronger nonlinear effects such as cross-phase modulation (XPM) and cross-gain modulation (XGM). These characteristics make CR-SOAs suitable for all-optical signal processing beyond 100 Gb/s [12]. When integrated into a Mach–Zehnder interferometer (MZI) configuration, CR-SOAs enable high-speed, low-power, and scalable logic operations due to the phase-sensitive interference properties of MZIs [13,14]. The CR-SOA-MZI combination has been shown to offer high extinction ratios and excellent signal integrity, rendering it a key component for realizing AOLGs [15,16,17,18,19,20,21]. In our previous work [21], we explored the use of CR-SOA-MZI architecture for designing a high-speed all-optical half-adder. That study focused specifically on implementing XOR and AND logic functions for basic arithmetic operations. In contrast, the present work expands upon that foundation and explores the potential of the same device configuration in a broader logical context. Specifically, we numerically demonstrate an all-optical encoder (in the form of one-hot logic minterm generation) and comparator system capable of operating at 120 Gb/s, which supports five essential logic operations: $\bar{A}B$, $A\bar{B}$, AB (AND), $\bar{A}\;\bar{B}$ (NOR), and AB + $\bar{A}\;\bar{B}$ (XNOR). It is important to note that the term “encoder” is used here in a broad sense to describe the generation of logical minterms (one-hot encoded outputs) from two binary inputs. This functionality corresponds directly to that of a standard 2-to-4 decoder in digital logic, where each output line represents a unique input condition. We have retained the term “encoder” to emphasize the mapping of binary inputs into distinguishable logic states, while also clarifying its equivalence to decoding behavior. This shift from arithmetic to comparative and encoding logic operations represents a new configuration and functionality, leveraging the same device in a novel and more complex application domain. Recent research has made significant progress in developing all-optical encoders and comparators using both conventional SOAs and quantum-dot SOAs (QD-SOAs) [22,23,24,25,26]. For instance, Komatsu et al. [22] demonstrated an ultrafast all-optical digital comparator based on QD-SOAs, achieving an enhanced speed and extinction ratio due to the discrete energy levels and fast carrier dynamics of quantum dots. Similarly, Kaur and Prakash [23] explored the nonlinear characteristics of conventional SOAs to implement logic-based comparator operations, emphasizing simplicity but with limited scalability and speed. Lei et al. [24] proposed a 40 Gb/s all-optical 4-bit priority encoder employing cross-gain modulation (XGM) in SOAs, offering high-speed operation but constrained by signal degradation at higher bit levels. Wang et al. [25] further enhanced system efficiency by simultaneously realizing both encoder and comparator functions at 40 Gb/s using SOAs, though integration complexity and noise were noted challenges. Additionally, Zhang et al. [26] presented a multifunctional setup capable of simultaneously operating as a digital comparator and a half-subtractor for 40 Gb/s DPSK signals, showcasing the versatility of SOA-based designs. Despite these advances, most existing designs are tailored for specific operations and face limitations in scalability, complexity, or multi-functionality, highlighting the need for more integrated, compact, and high-speed solutions. In this work, we present a comprehensive numerical analysis of a CR-SOA-MZI-based design capable of supporting simultaneous encoding and comparison operations at 120 Gb/s. We evaluate the system’s performance using the quality factor (QF) as a key performance metric and examine the influence of key operating parameters on signal integrity and logic accuracy. The calculated QF values for the five logic operations are as follows: 17.56 for $\bar{A}B$, 17.04 for $A\bar{B}$, 19.05 for AB (AND), 10.95 for $\bar{A}\;\bar{B}$ (NOR), and 8.33 for AB + $\bar{A}\;\bar{B}$ (XNOR). The design employs return-to-zero (RZ) modulation to preserve signal integrity at high bit rates [27,28]. By demonstrating multiple logic functions within a single platform, this study provides a scalable and multifunctional framework for future photonic computing systems. It contributes to the growing body of research aimed at integrating various logic functionalities into compact and efficient photonic circuits.

This paper is organized as follows: Section 2 introduces the CR-SOA, starting with Section 2.1, which explains the operational principles, detailing how the CR-SOA operates within the system to achieve the intended logic functions. Section 2.2 presents the numerical analysis, highlighting the calculations and simulations used to optimize the design and assess its performance. Section 3 describes the encoder and comparator, outlining their roles in encoding and comparing input signals for efficient optical logic operations. Section 4 discusses the results, providing a comprehensive evaluation of the system’s performance with key metrics such as the eye diagram, QF, and signal integrity at high data rates. Section 5 offers a comparison, contrasting the CR-SOA-based logic gate with other related SOA designs and emphasizing the advantages of our approach in terms of speed and reliability. Section 6 details the potential experimental setup, including the configuration and components used to validate the design and assess its practical performance. Finally, Section 7 concludes the paper by summarizing the key findings and highlighting the potential of CR-SOA-based logic gates for high-speed optical communication systems.

## 2. CR-SOA

### 2.1. Operation Principle

**Figure 1:**
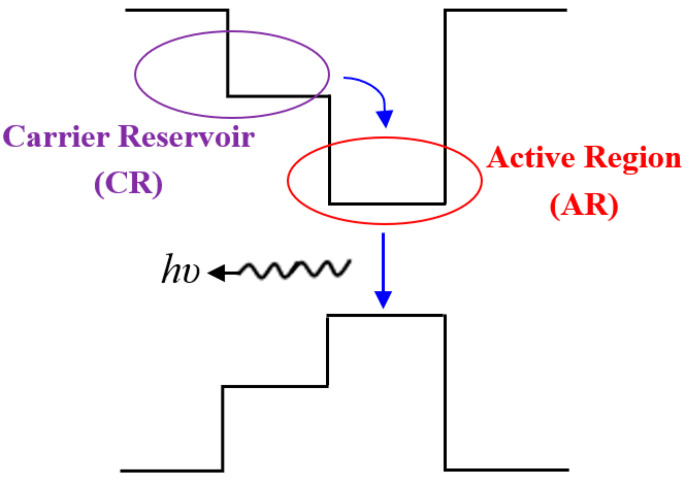
Carrier transition processes in CR-SOA, illustrating the movement of carriers between the carrier reservoir (CR) and the active region (AR) during operation.

The CR-SOA incorporates a carrier reservoir (CR) layer adjacent to the active region (AR), as shown in Figure 1 [15,18,21]. When a bias current is applied, carriers begin to fill the energy levels in both the AR and CR layers. At lower current levels, the quasi-Fermi level is closer to the AR band edge, leading to a higher carrier density in the AR compared to the CR. As the current increases, the quasi-Fermi level shifts upward, resulting in the CR becoming populated. This populated CR region then supplies carriers to the AR, replenishing those depleted by the input optical signal. The CR-SOA’s fast response is driven by the ultrafast carrier transition from the CR to the AR, with a typical transition time ranging from 5 to 10 ps [11,12]. This quick carrier replenishment allows for rapid gain and phase dynamics, similar to QD-SOAs [22,29,30]. The faster recovery times in CR-SOAs enhance the speed and efficiency of optical data processing, making them ideal for high-speed optical communication systems. Furthermore, CR-SOAs provide comparable performance to QD-SOAs but are more cost-effective and easier to fabricate, offering significant advantages in practical applications.

### 2.2. Numerical Analysis

The CR-SOA’s dynamic behavior is governed by time-dependent gain equations that account for carrier recombination between the active region (AR) and the carrier reservoir (CR), along with ultrafast intraband effects such as carrier heating (CH) and spectral hole burning (SHB). These effects are crucial in determining the device’s overall gain response and high-speed operation. The governing equations can be expressed as follows [11,12,15,18,21]:

(1) $$\frac{dh_{AR}(t)}{dt}\;\;=\;\;\frac{h_{CR}(t)-h_{AR}(t)}{\tau_{t}(1+\eta )}\;\;+\;\;\frac{\eta h_{0}}{\tau_{c}(1+\eta )}\;\;-\;\;\frac{h_{AR}(t)}{\tau_{c}}\;\;-\;\;(exp\;[h_{AR}(t)\;\;+\;\;h_{CH}(t)\;\;+\;\;h_{SHB}(t)]\;\;-\;\;1)\;\;\frac{P_{in,\;\;CR-SOA}(t)}{E_{sat}}$$

(2) $$\frac{dh_{CR}(t)}{dt}\;\;=\;\;-\frac{\eta (h_{CR}(t)-h_{AR}(t)}{\tau_{t}(1+\eta )}\;\;+\;\;\frac{h_{0}-h_{CR}(t)}{\tau_{c}(1+\eta )}\;\;-\;\;\frac{h_{CR}(t)}{\tau_{c}}$$

(3) $$\frac{dh_{CH}(t)}{dt}\;\;=\;\;-\frac{h_{CH}(t)}{\tau_{CH}}\;\;-\;\;\frac{\varepsilon_{CH}}{\tau_{CH}}(exp\;[h_{AR}(t)\;\;+\;\;h_{CH}(t)\;\;+\;\;h_{SHB}(t)]\;\;-\;\;1)\;P_{in,\;\;CR-SOA}(t)$$

(4) $$\frac{dh_{CH}(t)}{dt}\;\;=\;\;-\frac{h_{CH}(t)}{\tau_{CH}}\;\;-\;\;\frac{\varepsilon_{CH}}{\tau_{CH}}(exp\;[h_{AR}(t)\;\;+\;\;h_{CH}(t)\;\;+\;\;h_{SHB}(t)]\;\;-\;\;1)\;P_{in,\;\;CR-SOA}(t)$$

where

- τ_t_: Transition lifetime from CR to AR
- τ_c_: Carrier lifetime
- η: Population inversion factor, defined as the ratio of carrier densities in the active region (AR) and carrier reservoir (CR), i.e., η = N_AR_/N_CR_.
- τ_CH_: Temperature relaxation rate due to CH
- τ_SHB_: Temperature relaxation rate due to SHB
- ε_CH_: Nonlinear gain suppression factor due to CH
- ε_SHB_: Nonlinear gain suppression factor due to SHB

The total gain of the CR-SOA is determined by contributions from the AR recombination, CH, and SHB, represented as

(5) $$G_{CR-SOA_{i}}(t)\;\;=\;\;exp\;[h_{AR}(t)\;\;+\;\;h_{CH}(t)\;\;+\;\;h_{SHB}(t)],\;\;\;\;\;i=1,2,\ldots \ldots$$

The unsaturated power gain, G_0_, is given by

(6) $$G_{0}=exp[h_{0}]\;\;=\;\;\Gamma \alpha \;\;\left(\frac{I\tau_{c}}{eV}\;\;-N_{tr}\right)L$$

where

- Γ: Optical confinement factor
- α: Differential gain
- N_tr_: Transparency carrier density
- I: Injection current
- e: Electron charge
- V = wdL: Active layer volume, where w is the width, d is the thickness, and L is the length of the active region.

The saturation energy E_sat_ is defined as

(7) $$E_{sat}=P_{sat}\;\tau_{c}=\;\frac{wdh\upsilon }{\Gamma \alpha }$$

where

- P_sat_: Saturation power
- h: Planck’s constant
- υ: Signal frequency

For numerical simulations, the input optical power pulses P_in,CR−SOA_(t) are modeled as Gaussian-shaped pulses within a pseudorandom binary sequence (PRBS) of length N, with energy E_0_, full-width at half maximum (FWHM) τ_FWHM_, and bit period T:

(8) $$P_{A,\;B}(t)\;\;\equiv P_{in,\;CR-SOA}(t)=\;\sum_{n\;=\;1}^{N}a_{n(A,B)}\frac{2\sqrt{\ln[2}]\;E_{0}}{\sqrt{\pi }\;\tau_{FWHM}}\;\;\exp\left[-\frac{4\ln[2]{\left(t\;\;-\;\;nT\right)}^{2}}{\tau_{FWHM}^{2}}\right]$$

where a_n(A,B)_ represents the PRBS data streams for inputs A and B, with each taking values of ‘0’ or ‘1’.

The induced phase shift in the CR-SOA is given by

(9) $$\Phi_{CR-SOA_{i}}(t)\;\;=\;\;-\;\;0.5\;\;\left(\alpha \;h_{AR}(t)\;\;+\;\;\alpha_{CH}\;h_{CH}(t)\right),\;\;\;i=1,2,\ldots$$

where α is the traditional linewidth enhancement factor (α-factor) and α_CH_ is the linewidth enhancement factor for CH. The SHB contribution to phase shift is negligible, i.e., α_SHB_ ≈ 0 [15,18,21].

This set of equations provides a comprehensive numerical framework for analyzing the CR-SOA’s dynamic gain and phase response under high-speed optical signal modulation. By incorporating carrier dynamics, intraband effects, and gain suppression mechanisms, these equations enable precise modeling of CR-SOA performance in optical logic and communication applications.

In this study, the quality factor (QF) is employed to evaluate the performance of the optical functions under investigation. The QF is a crucial metric for assessing the signal quality in optical systems, as it directly correlates with the bit error rate (BER) [31], a key performance indicator in communication systems. The QF quantifies the response of an optical circuit to the entire duration of the input data patterns, providing insight into its performance when the system operates under heavy stress. This is particularly useful in scenarios where the system’s operation is pushed to its limits, as it helps assess potential performance degradation. The QF is defined as [11]:

(10) $$QF=\frac{P_{1}-P_{0}}{\sigma_{1}+\sigma_{0}}$$

where P_1_ and P_0_ represent the mean peak powers of the signal at logic “1” and “0” levels, respectively, while σ_1_ and σ_0_ are the standard deviations of the noise at those levels. This definition enables a direct assessment of the signal-to-noise ratio, which is essential for determining the reliability of the system in high-speed operations. Importantly, there exists a well-established exponential relationship between the QF and the BER in optical systems, typically approximated as [11,31]:

(11) $$BER=\frac{1}{\sqrt{2\pi }}\frac{\exp[-QF^{2}/2]}{QF}$$

This equation shows that as the QF increases, the BER decreases rapidly. For example, a QF value of 6 corresponds to a BER of approximately 10^−9^, which is a standard benchmark for error-free operation in high-speed optical communication systems [15,18,21]. Therefore, monitoring the QF allows for an indirect yet highly effective estimation of BER and is widely used in evaluating the integrity of optical signal transmission. While several definitions of the QF exist in optical system analysis, including those based on eye diagrams or frequency-domain measures such as FWHM of resonances [32,33], this study uses the time-domain QF definition, as it provides a direct correlation with BER and is widely applied in digital optical communication systems [34,35,36,37]. To obtain accurate results, the Adams numerical method is employed in Wolfram Mathematica^®^ to solve all time-dependent equations. This method is known for its efficiency in solving complex differential equations, making it an ideal approach for the detailed analysis of optical systems over time. By integrating the QF as the performance criterion and utilizing Mathematica for precise numerical analysis, this study provides a robust evaluation of the optical logic gate’s functionality, addressing both the system’s performance and the potential impact of noise and distortion on the overall signal integrity. The simulations use the default numerical parameters listed in Table 1 [11,12,15,16,17,18,19,20,21,22,23,24,25,26], which are consistent with the previous literature on SOA- and CR-SOA-based optical logic devices. Many of these values, including carrier lifetimes, linewidth enhancement factors, saturation powers, and SOA geometrical dimensions, were selected based on experimental works and validated simulation studies [11,12,15,18,21,22,23,24,25,26]. Notably, the pulse energy (E_0_), set at 0.7 pJ, lies within the typical operating range used in similar high-speed optical logic circuits [15,16,17]. To ensure robustness, we performed additional simulations by varying the pulse energy between 0.5 pJ and 1 pJ, observing only minor variations in the QF (<5%) while maintaining error-free operation (BER < 10^−9^), indicating the resilience of the design. Similarly, the wavelengths of signal A (1550.7 nm) and signal B (1549.3 nm) were carefully chosen to match the gain bandwidth of the SOA and to enable efficient four-wave mixing and cross-phase modulation within the interferometer arms. These choices are aligned with values reported in recent CR-SOA-based logic gate studies [15,16,17,18,19,20], where close channel spacing (∼1–2 nm) within the C-band is employed to minimize dispersion and achieve high-speed operation. We also tested the impact of a ± 0.5 nm shift in input wavelengths and confirmed that the performance remained stable without significant degradation in the QF.

## 3. Encoder and Comparator

**Figure 2:**
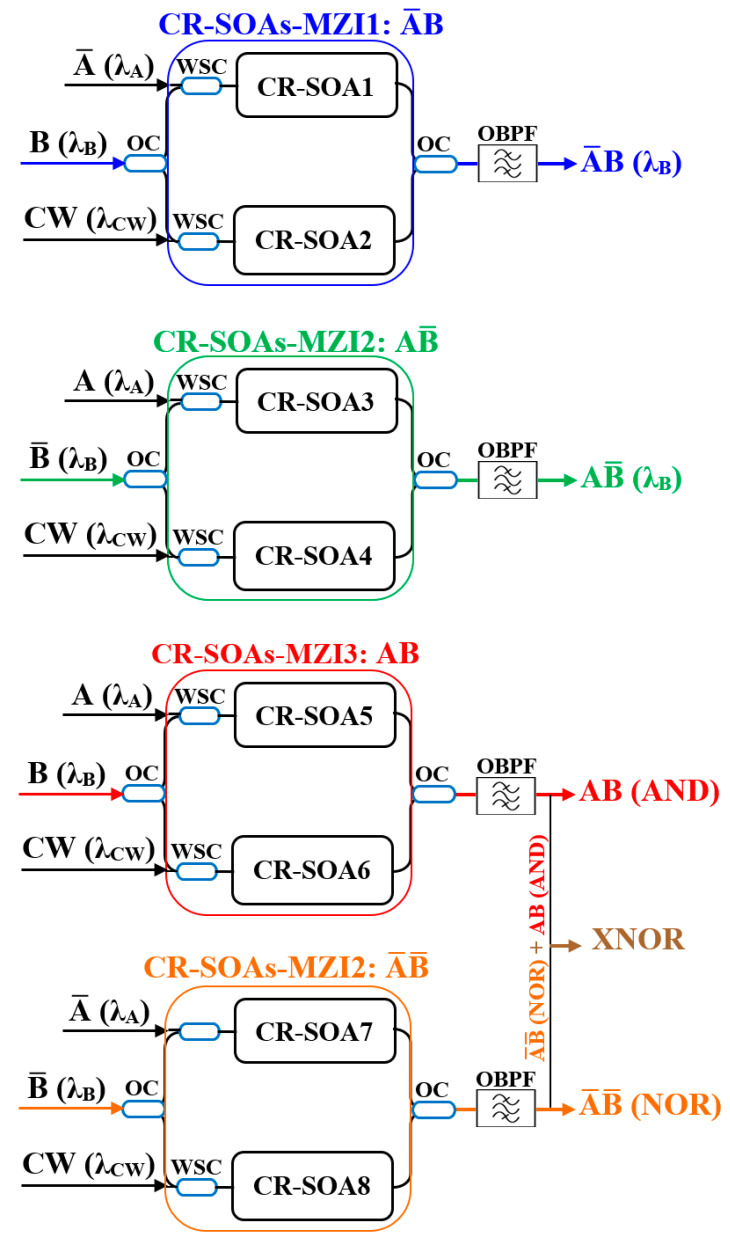
Operation principle of the all-optical encoder and comparator using CR-SOA-MZIs.

The operation principle of the all-optical encoder and comparator using a CR-SOA-MZI relies on designing, executing, and combining five essential logic operations—$\bar{A}B$, $A\bar{B}$, AB (AND), $\bar{A}\;\bar{B}$ (NOR), and AB + $\bar{A}\;\bar{B}$ (XNOR)—using four CR-SOA-MZIs, as shown in Figure 2. The system involves three inputs: signals A, B, and a continuous wave (CW), injected into the CR-SOA-MZIs to achieve the desired logic functions. The wavelengths for signals A, B, and CW are 1550.7 nm, 1549.3 nm, and 1551.3 nm, respectively [25]. The CW signal plays a crucial role by providing a constant reference power level, which enables efficient cross-gain modulation (XGM) effects for the logic operations. In the encoder, each of the logic outputs corresponds to a different input condition: $\bar{A}B$, $A\bar{B}$, AB, and $\bar{A}\;\bar{B}$. The CR-SOA-MZIs perform the logic operations for these outputs. In the CR-SOA-MZI1, the $\bar{A}B$ operation is realized at wavelength λ_B_, with the optical power of signal A being significantly larger than signal B, aided by the CW signal at λ_CW_, optimizing the XGM effect. Similarly, the CR-SOA-MZI2 performs the $A\bar{B}$ operation, where signal B holds higher optical power than A, and the CW signal further enhances the XGM effect. The AND (AB) operation is realized in the CR-SOA-MZI3, where the combined signals A, B, and CW generate the output at wavelength λB. The NOR operation ($\bar{A}\;\bar{B}$) is executed in the CR-SOA-MZI4, where an AND operation between $\bar{A}\;\bar{B}$ is carried out, supported by the CW signal and the XGM effect. Lastly, the XNOR gate (AB + $\bar{A}\;\bar{B}$) is achieved by combining the outputs of the AND and NOR gates, producing the logic function AB + $\bar{A}\;\bar{B}$. For the comparator, the system provides three outputs to represent the results of comparing signals A and B. The A < B output is generated when A is bit “0” and B is bit “1”, corresponding to the AB operation. When both A and B are either “0” or “1”, the A = B output is bit “1”, represented by $\bar{A}\;\bar{B}$ or XNOR logic. Finally, when A is bit “1” and B is bit “0”, the A > B output is activated, represented by the AB operation. To manage the input signals effectively, 3 dB optical couplers (OCs) distribute the A, B, and CW signals to the respective CR-SOA-MZIs, ensuring optimal power levels. Wavelength-selective couplers (WSCs) direct the signals to the correct MZIs based on their wavelengths, facilitating each logic function at its designated wavelength. Optical bandpass filters (OBPFs) at the output of each CR-SOA-MZI ensure that only the desired logic gate outputs are selected, eliminating unwanted signals and preserving high signal integrity. The combination of OCs, WSCs, and OBPFs ensures efficient generation and extraction of optical logic operations, providing a robust solution for high-speed all-optical digital encoders and comparators, making it a promising approach for future optical computing and photonic logic circuits. The truth table for both the encoder and comparator are shown in Table 2, which clarifies the logic outputs corresponding to various input combinations.

To numerically implement the $\bar{A}B$ operation, the optical input powers fed into CR-SOA1 and CR-SOA2 of the CR-SOA-MZI1 can be represented as

(12) $$P_{in,\;\;CR-SOA_{1}}(t)\;\;=\;\;P_{\bar{A}}(t)\;\;+\;\;0.5\;P_{B}(t)$$

(13) $$P_{in,\;\;CR-SOA_{2}}(t)\;\;=\;\;P_{CW}\;+\;\;0.5\;P_{B}(t)$$

Then, the output power for the $\bar{A}B$ operation in the CR-SOA-MZI1 is expressed as [11]

(14) $$P_{\bar{A}B}(t)=\;\;0.25\;P_{B}(t)\;\left(G_{CR-SOA_{1}}(t)\;\;+\;\;G_{CR-SOA_{2}}(t)\;\;-\;\;2\sqrt{G_{CR-SOA_{1}}(t)\;\;G_{CR-SOA_{2}}(t)}\;\;cos\;[\Phi_{CR-SOA_{1}}(t)\;\;-\;\;\Phi_{CR-SOA_{2}}(t)]\right)$$

To perform the $A\bar{B}$ operation, the input optical powers applied to CR-SOA3 and CR-SOA4 in CR-SOA-MZI2 are defined as

(15) $$P_{in,\;\;CR-SOA_{3}}(t)\;\;=\;\;P_{A}(t)\;\;+\;\;0.5\;P_{\bar{B}}(t)$$

(16) $$P_{in,\;\;CR-SOA_{4}}(t)\;\;=\;\;P_{CW}\;+\;\;0.5\;P_{\bar{B}}(t)$$

Consequently, the $A\bar{B}$ output power of the CR-SOA-MZI2 is determined by

(17) $$P_{A\bar{B}}(t)=\;\;0.25\;P_{\bar{B}}(t)\;\left(G_{CR-SOA_{3}}(t)\;\;+\;\;G_{CR-SOA_{4}}(t)\;\;-\;\;2\sqrt{G_{CR-SOA_{3}}(t)\;\;G_{CR-SOA_{4}}(t)}\;\;cos\;[\Phi_{CR-SOA_{3}}(t)\;\;-\;\;\Phi_{CR-SOA_{4}}(t)]\right)$$

For the AB (AND) operation, the optical power inputs directed into CR-SOA5 and CR-SOA6 of CR-SOA-MZI3 can be defined as

(18) $$P_{in,\;\;CR-SOA_{5}}(t)\;\;=\;\;P_{A}(t)\;\;+\;\;0.5\;P_{B}(t)$$

(19) $$P_{in,\;\;CR-SOA_{6}}(t)\;\;=\;\;P_{CW}\;+\;\;0.5\;P_{B}(t)$$

Then, the output power for the AB (AND) operation at the CR-SOA-MZI3 is expressed as

(20) $$P_{AB}(t)=\;\;0.25\;P_{B}(t)\;\left(G_{CR-SOA_{5}}(t)\;\;+\;\;G_{CR-SOA_{6}}(t)\;\;-\;\;2\sqrt{G_{CR-SOA_{5}}(t)\;\;G_{CR-SOA_{6}}(t)}\;\;cos\;[\Phi_{CR-SOA_{5}}(t)\;\;-\;\;\Phi_{CR-SOA_{6}}(t)]\right)$$

To perform the $\bar{A}\;\bar{B}$ (NOR) operation, the input optical powers fed into CR-SOA7 and CR-SOA8 of the CR-SOA-MZI4 are represented as

(21) $$P_{in,\;\;CR-SOA_{7}}(t)\;\;=\;\;P_{\bar{A}}(t)\;\;+\;\;0.5\;P_{\bar{B}}(t)$$

(22) $$P_{in,\;\;CR-SOA_{8}}(t)\;\;=\;\;P_{CW}\;+\;\;0.5\;P_{\bar{B}}(t)$$

Then, the $\bar{A}\;\bar{B}$ (NOR) output power generated by the CR-SOA-MZI4 is determined as

(23) $$P_{\bar{A}\bar{B}}(t)=\;\;0.25\;P_{\bar{B}}(t)\;\left(G_{CR-SOA_{7}}(t)\;\;+\;\;G_{CR-SOA_{8}}(t)\;\;-\;\;2\sqrt{G_{CR-SOA_{7}}(t)\;\;G_{CR-SOA_{8}}(t)}\;\;cos\;[\Phi_{CR-SOA_{7}}(t)\;\;-\;\;\Phi_{CR-SOA_{8}}(t)]\right)$$

The XNOR output power is obtained by combining the results of the AB and $\bar{A}\;\bar{B}$ operations, as expressed in [38]:

(24) $$P_{XNOR}(t)=\;\;P_{AB}(t)\;\;+\;\;P_{\bar{A}\;\bar{B}}(t)$$

## 4. Results

**Figure 3:**
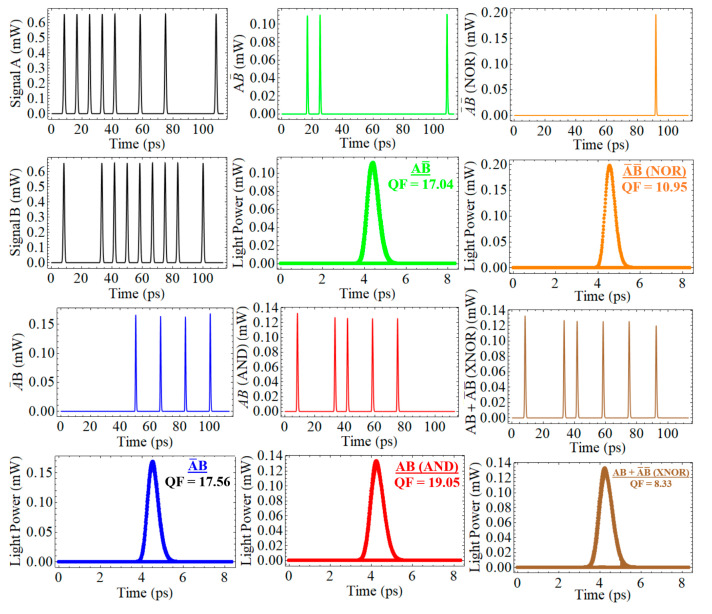
Performance of the all-optical encoder and comparator using CR-SOA-MZI at 120 Gb/s, executing five logic operations with high QFs: 17.56 ($\bar{A}B$), 17.04 ($A\bar{B}$), 19.05 (AND), 10.95 (NOR), and 8.33 (XNOR).

The performance of the proposed all-optical encoder and comparator using an CR-SOA-MZI at 120 Gb/s was evaluated with input signals A and B, successfully performing five distinct logic operations: $\bar{A}B$, $A\bar{B}$, AB (AND), $\bar{A}\;\bar{B}$ (NOR), and AB + $\bar{A}\;\bar{B}$ (XNOR). The output logic functions demonstrated exceptionally high QFs, with values of 17.56 for $\bar{A}B$, 17.04 for $A\bar{B}$, 19.05 for AB (AND), 10.95 for $\bar{A}\;\bar{B}$ (NOR), and 8.33 for XNOR, as shown in Figure 3. These high QFs are attributed to the efficient XGM effects enabled by the CR-SOA-MZI configuration. The CR-SOA’s nonlinearity ensures strong interactions between the signals, resulting in enhanced signal modulation and high-speed logic operations. However, the relatively lower QF of 8.33 for the XNOR operation can be explained by the increased complexity of combining two different logic gates (AND and NOR) to achieve the XNOR output. This operation involves a higher level of interaction and interference between signals, which can lead to slight distortions in the output signal. Additionally, the combined nature of the XNOR function may cause suboptimal signal reinforcement compared to individual logic operations, resulting in higher noise levels and less efficient modulation. As a result, this leads to a lower QF for the XNOR output. The eye diagrams for all the logic operations were open and clear, confirming the high signal integrity and minimal distortion across the outputs, except for the XNOR function, which exhibited some mild distortion. These results highlight the excellent performance and reliability of the CR-SOA-MZI configuration for most logic operations, with the XNOR operation showing a slight reduction in quality, which could be further optimized in future designs.

**Figure 4:**
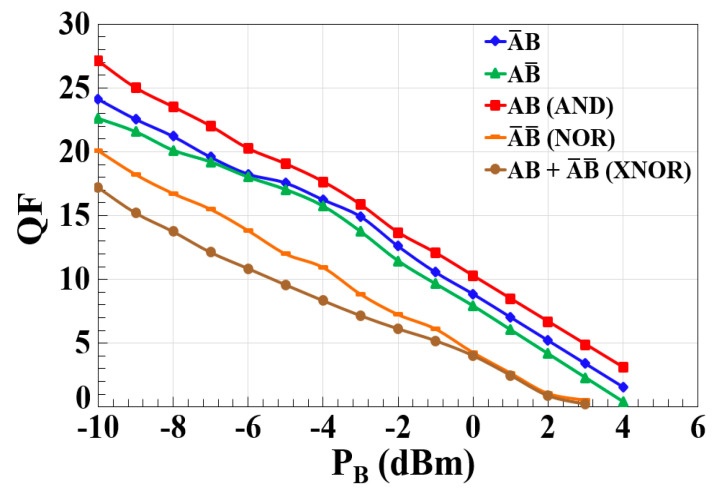
Simulation results showing the variation in the QF for different output logic operations of the encoder and comparator ($\bar{A}B$, $A\bar{B}$, AB (AND), $\bar{A}\;\bar{B}$ (NOR), and AB + $\bar{A}\;\bar{B}$ (XNOR)) as a function of the power of signal B (PB) using CR-SOA-MZI at 120 Gb/s.

The simulation results for various optical logic operations, including $\bar{A}B$, $A\bar{B}$, AB (AND), $\bar{A}\;\bar{B}$ (NOR), and AB + $\bar{A}\;\bar{B}$ (XNOR), in the CR-SOA-MZI configuration, reveal the crucial impact of the power of signal B (PB) on the overall performance of AOLGs. As shown in Figure 4, the data demonstrate a clear inverse relationship between the power of signal B and the QF of the output logic functions. At lower input powers, such as −10 dBm, the interaction between signals A and B is more effectively managed, leading to higher QFs. However, as the power of signal B increases, the CR-SOA experiences saturation, diminishing the XGM effect, which is essential for maintaining the logic operations’ quality. In the $\bar{A}B$ operation, for example, an initial QF of 24.12 is observed at –10 dBm, but this value decreases as signal B’s power rises, indicating that the cross-gain modulation becomes less efficient at higher powers. Similar trends are seen in the $A\bar{B}$ and AB operations, where the power of signal B plays a dominant role in the modulation process, and excessive power results in over-saturation, reducing modulation depth and signal clarity. Notably, the NOR operation ($\bar{A}\;\bar{B}$) exhibits extreme sensitivity to the power of signal B, with a significant drop in the QF reaching near zero at 4 dBm. This highlights the complex interactions between signals A and B within the SOA, where higher power levels lead to inefficient carrier density modulation and signal distortion. The XNOR operation, which combines both AND and NOR gates, also demonstrates a drastic reduction in the QF as signal B’s power increases, indicating that multi-gate logic operations are particularly susceptible to degradation when signal B’s power is excessive, leading to interference between the gates. We attribute the reduction in the QF at higher input powers primarily to CR-SOA saturation, which leads to amplitude imbalance and reduced differential gain between the MZI arms. Additionally, pattern-dependent nonlinearities may further degrade signal quality under high-power and high-speed conditions, although saturation appears to be the dominant factor. These results emphasize the necessity of precisely managing the power of signal B to maintain optimal performance in all-optical logic gate designs. The observed trends, as shown in Figure 4, suggest that there is an optimal power range for signal B, where the XGM effect is maximized without introducing detrimental saturation effects. For high-speed optical communication systems, power optimization strategies, such as adaptive power adjustment or feedback mechanisms, are crucial to ensure that both input signals remain within their optimal power levels. At data rates exceeding 120 Gb/s, the primary limiting factor is expected to be the carrier recovery time of the CR-SOA rather than the bandwidth of the MZI, which is inherently broad due to its passive nature. As bit intervals become shorter, the slow recovery of carrier density can lead to patterning effects, reduced modulation efficiency, and signal distortion. Future work should explore faster SOA structures (e.g., QD-SOAs) or hybrid material systems to overcome this limitation. Further simulations that vary the power conditions for both signals A and B could provide additional insights into the ideal power levels for different logic operations, ultimately improving the performance and efficiency of CR-SOA-MZI-based systems. This study underlines the importance of careful power management to avoid saturation, ensuring that the logic gates deliver a high QF and maintain efficient signal processing, particularly in systems that require high-speed and reliable operation for advanced optical communication technologies. Future research should focus on exploring optimal power ranges for various logic operations and developing techniques to stabilize performance across a range of operational conditions.

**Figure 5:**
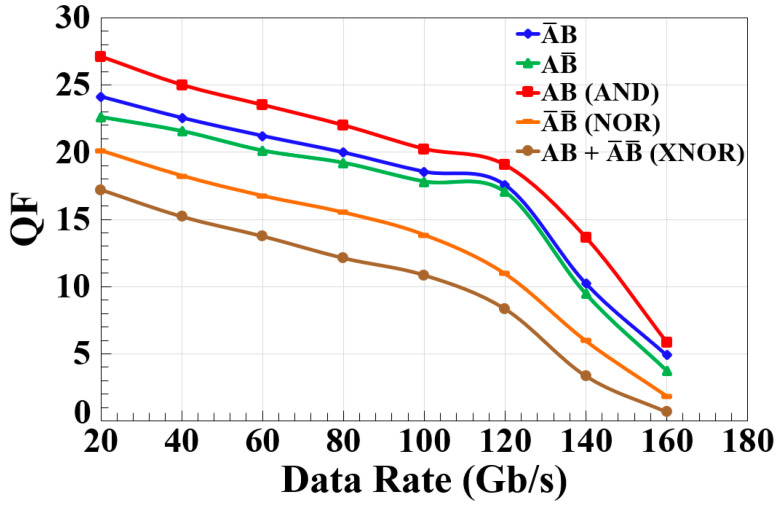
Simulation results showing the variation in the QF for different output logic operations of the encoder and comparator ($\bar{A}B$, $A\bar{B}$, AB (AND), $\bar{A}\;\bar{B}$ (NOR), and AB + $\bar{A}\;\bar{B}$ (XNOR)) as a function of data rate using CR-SOA-MZI at 120 Gb/s.

The results, as shown in Figure 5, illustrate the variation in the QF against the data rate, using the CR-SOA-MZI for different logic gate operations: $\bar{A}B$, $A\bar{B}$, AB (AND), $\bar{A}\;\bar{B}$ (NOR), and AB + $\bar{A}\;\bar{B}$ (XNOR). The QF decreases for all logic operations as the data rate increases from 20 Gb/s to 160 Gb/s. For instance, for $\bar{A}B$, the QF drops from 24.12 at 20 Gb/s to 4.92 at 160 Gb/s, while for $A\bar{B}$, it decreases from 22.61 to 3.75. A similar trend is observed in the AND operation (AB), where the QF decreases from 27.12 at 20 Gb/s to 5.85 at 160 Gb/s, indicating a gradual degradation in performance with increasing data rates. The NOR operation ($\bar{A}\;\bar{B}$) follows the same pattern, with the QF declining from 20.12 to 1.85, and the XNOR operation (AB + $\bar{A}\;\bar{B}$) experiences a sharper decrease from 17.21 to 0.65. This decline in the QF as the data rate increases suggests that higher-speed operations impose limitations on the system’s ability to maintain strong functional performance, likely due to factors such as increased signal distortion, carrier recovery limitations, or phase noise accumulation in the optical setup. The decreasing trend across all logic gates indicates that as the data rate increases, system impairments, such as gain saturation in CR-SOA-MZI configurations, become more pronounced, leading to signal degradation. The more significant drop in XNOR performance compared to other logic functions might be attributed to its reliance on both AND and NOR operations, where the cumulative effects of signal degradation have a greater impact on its overall efficiency. Additionally, the AND function maintains the highest QF values across all data rates, while XNOR consistently shows the lowest, highlighting a stronger resilience of the AND operation in high-speed conditions. These results suggest that optimizing parameters such as the injected optical power, bias current of the CR-SOA, or phase tuning in the interferometer could mitigate performance losses at higher data rates. Further analysis could focus on investigating alternative configurations or signal processing techniques to enhance the robustness of AOLGs operating at ultra-high speeds.

**Figure 6:**
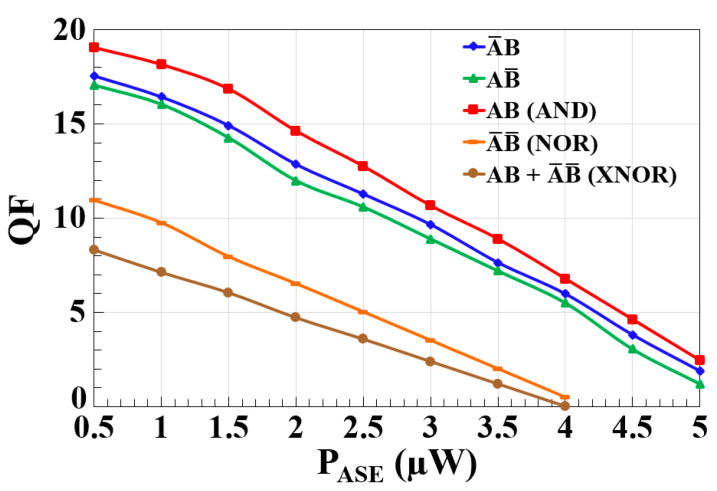
Simulation results showing the variation in the QF for different output logic operations of the encoder and comparator ($\bar{A}B$, $A\bar{B}$, AB (AND), $\bar{A}\;\bar{B}$ (NOR), and AB + $\bar{A}\;\bar{B}$ (XNOR)) as a function of amplified spontaneous emission power (P_ASE_) using CR-SOA-MZI at 120 Gb/s.

The results illustrate the variation in the QF as a function of amplified spontaneous emission (ASE) power (PASE) for different logic gate operations—$\bar{A}B$, $A\bar{B}$, AB (AND), $\bar{A}\;\bar{B}$ (NOR), and AB + $\bar{A}\;\bar{B}$ (XNOR)—using a CR-SOA-MZI-based system at 120 Gb/s, as shown in Figure 6. PASE is added numerically to the logic operations’ output powers given by Equations (14), (17), (20) and (23), ensuring a realistic evaluation of ASE-induced degradation in system performance. The ASE power is calculated using the following equation [39]:

(25) $$P_{ASE}=\;\;N_{sp}\;\left(G-1\right)h\nu B_{0}$$

where

- N_sp_: Spontaneous emission factor
- G: Signal gain
- B_0_: Optical bandwidth

As P_ASE_ increases from 0.5 μW to 5 μW, a significant reduction in the QF is observed for all logic functions. At P_ASE_ = 0.5 μW, the highest QF is recorded for the AND operation (19.05), followed by $\bar{A}B$ (17.56) and $A\bar{B}$ (17.04). The NOR and XNOR operations show lower QF values of 10.95 and 8.33, respectively. This trend indicates that the AND function maintains the strongest resilience against initial ASE-induced impairments, while XNOR is the most sensitive to ASE-related performance degradation. With increasing P_ASE_, the QF decreases progressively. At P_ASE_ = 2 μW, the QF for $\bar{A}B$, $A\bar{B}$, and AND drops to 12.86, 11.98, and 14.62, respectively, representing a significant performance decline. NOR and XNOR gates experience more severe degradation, with QF values falling to 6.52 and 4.72, respectively. This suggests that interference-based logic operations, such as NOR and XNOR, are more affected by ASE due to phase noise accumulation and loss of contrast in constructive and destructive interference mechanisms. At P_ASE_ = 4 μW, NOR and XNOR operations approach near-failure states, with QF values plunging to 0.493 and 0.007, respectively. Meanwhile, the AND gate retains some level of performance with a QF of 6.78, demonstrating relatively better tolerance to ASE. The rapid deterioration of XNOR performance at high ASE levels suggests that its dependence on both AND and NOR operations compounds its susceptibility to noise. At the highest P_ASE_ levels (4.5 μW and 5 μW), QF values for all logic gates drop drastically, with the AND gate falling to 2.45, while $\bar{A}B$ and $A\bar{B}$ decline to 1.88 and 1.21, respectively. The NOR and XNOR functions become completely non-functional, highlighting their extreme sensitivity to ASE. These results, as shown in Figure 6, emphasize the significant impact of ASE on CR-SOA-MZI-based logic operations at ultra-high data rates. The AND operation demonstrates the highest resilience, while XNOR is the most vulnerable. The rapid decline in the QF with increasing ASE suggests that mitigating ASE is crucial for maintaining the performance of AOLGs. Future improvements could focus on optimizing SOA gain dynamics, employing ASE suppression techniques, and refining phase stabilization methods to enhance the robustness of these logic gates in high-speed optical computing and communication applications.

## 5. Comparison

Table 3 systematically compares encoder and comparator designs employing quantum dot (QD-SOA), conventional (SOA), and carrier reservoir (CR-SOA) semiconductor optical amplifiers across 10–160 Gb/s data rates, with particular attention to extinction ratio (ER) and quality factor (QF) metrics. The QD-SOA comparator [22] establishes performance benchmarks, achieving 10 dB ER and 9.0 QF at 160 Gb/s through simulation, demonstrating exceptional signal integrity for ultra-high-speed applications. In contrast, the SOA-based comparator [23] operates at 10 Gb/s with slightly lower ER (9 dB) and lacks QF data, reflecting relaxed performance requirements at lower speeds. Experimental studies using SOA technology at 40 Gb/s [24,25,26] consistently achieve 10 dB ER (with [24] and [25] focusing on encoder designs, while [26] examines comparators), though the absence of QF reporting limits comprehensive performance evaluation. Notably, our CR-SOA implementation advances the field by operating at 120 Gb/s while maintaining 10 dB ER and a competitive QF (8.33–9.05), representing only a 7.4–11.1% reduction compared to the QD-SOA benchmark despite 25% higher speeds than 40 Gb/s implementations. The CR-SOA results reveal three key characteristics: (1) maintained ER parity with top-performing designs, (2) a slightly reduced but stable QF (8.33–9.05) at elevated speeds, and (3) clear optimization pathways through phase tuning, injection power adjustment, and noise reduction. This analysis yields important insights: QD-SOA maintains superior signal quality (QF = 9.0) at extreme speeds (160 Gb/s), while CR-SOA demonstrates excellent speed–QF compromise (8.33–9.05 at 120 Gb/s). The consistent ER = 10 dB across multiple studies [24,25,26] suggests reliable signal contrast at moderate speeds, though the prevalent lack of QF reporting (83% of compared SOA studies) represents a significant literature gap for comprehensive performance evaluation.

## 6. Potential Experimental Setup

**Figure 7:**
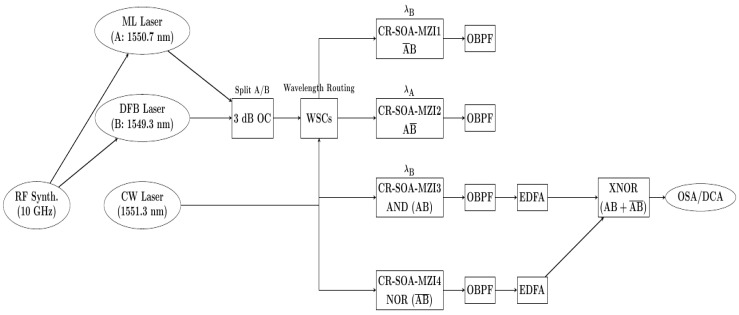
Envisioned experimental setup for the all-optical encoder and comparator using CR-SOA-MZIs.

The envisioned experimental setup for the all-optical encoder and comparator [24,25,26] using a CR-SOA-MZI is designed to perform five essential logic operations—$\bar{A}B$, $A\bar{B}$, AB (AND), $\bar{A}\;\bar{B}$ (NOR), and AB + $\bar{A}\;\bar{B}$ (XNOR)—using four CR-SOA-MZIs. A schematic representation of this proposed experimental configuration is illustrated in Figure 7. The system is driven by three input signals: A, B, and CW. The data signals A and B are generated using two synchronized laser systems, a rational harmonic mode-locked (ML) laser and a gain-switched distributed feedback (DFB) laser, producing ultra-short pulses with widths of 3.5 ps at repetition rates of 20 GHz and 40 GHz, respectively. A shared 10 GHz RF synthesizer synchronizes these lasers. The CW signal, operating at a wavelength of 1551.3 nm, is used as the reference power to enable efficient XGM in the CR-SOAs, enhancing the logic operations. In the encoder setup, the logic operations $\bar{A}B$, $A\bar{B}$, AB, and $\bar{A}\;\bar{B}$ are performed by four CR-SOA-MZIs, each optimized to handle different logic conditions. The input signals A and B, at wavelengths 1550.7 nm and 1549.3 nm, respectively, are injected into the MZIs along with the CW signal. A 3 dB OC splits the input signals A and B, directing them to the appropriate MZI through WSCs, which ensure the signals are routed based on their wavelength. While the current configuration operates with wavelengths centered around 1549.3–1551.3 nm, the setup is adaptable to a range of wavelengths within the C-band (1530–1565 nm). The optical components in the setup, such as the WSCs, OBPFs, and CR-SOAs, are tunable or can be replaced with versions that support different wavelengths. This flexibility allows for compatibility with wavelength-division multiplexing (WDM) systems, enabling multi-channel optical logic processing. Additionally, the system can be adapted to use wavelengths outside the C-band, depending on the specific application, making it versatile for different communication and computing technologies. In the CR-SOA-MZI1, the $\bar{A}B$ operation is realized at wavelength λ_B_, with signal A being significantly more powerful than signal B, aided by the CW signal at λ_CW_ to enhance the XGM effect. Similarly, the CR-SOA-MZI2 performs the $A\bar{B}$ operation, where signal B holds higher optical power than signal A, again supported by the CW signal to maximize the XGM effect. The AND operation (AB) is realized in the CR-SOA-MZI3, where the combined signals A, B, and CW generate the output at λ_B_. The NOR operation ($\bar{A}\;\bar{B}$) is executed in the CR-SOA-MZI4, where the XGM effect enables the logic operation. For the XNOR gate (AB + $\bar{A}\;\bar{B}$), the outputs from the AND and NOR gates are combined. OBPFs with narrow bandwidths are placed at the output of each CR-SOA-MZI to select only the desired logic gate output, eliminating any noise or unwanted signals. The output signals are further amplified using erbium-doped fiber amplifiers (EDFAs) to maintain the desired power levels for signal analysis. To assess the system’s performance, an optical spectrum analyzer (OSA) measures the optical spectrum and signal quality, while a digital communications analyzer (DCA) evaluates the output waveforms, providing insights into timing, logic levels, and signal integrity. Metrics such as the QF are used to verify the precision and efficiency of the logic gates. The system operates at data rates as high as 120 Gb/s, making it a promising solution for high-speed, all-optical digital encoders and comparators in optical computing and photonic logic circuits.

## 7. Conclusions

This study demonstrates a high-speed all-optical encoder and comparator operating at 120 Gb/s using a carrier reservoir semiconductor optical amplifier–Mach–Zehnder interferometer (CR-SOA-MZI), contributing to the ongoing development of advanced optical logic architectures. The distinct contributions of this work include the following: (1) the integration of encoding and comparison functionalities within a unified CR-SOA-MZI structure, enabling multifunctional operation without increased structural complexity; (2) the realization of five key logic functions—$\bar{A}B$, $A\bar{B}$, AB (AND), $\bar{A}\;\bar{B}$ (NOR), and AB + $\bar{A}\;\bar{B}$ (XNOR)—within a single platform operating at 120 Gb/s; (3) the use of quality factor (QF) analysis as a robust performance metric, with QF values exceeding 8, indicating strong signal fidelity even at ultra-high speeds; and (4) an in-depth numerical investigation examining how key system parameters—such as input power, data rate, and amplified spontaneous emission (ASE) noise—affect performance. The findings suggest that basic logic operations (e.g., AND, NOR) maintain strong performance stability, while more complex functions (e.g., XNOR) remain within acceptable operational thresholds. Compared with conventional SOA-MZI designs, the CR-SOA-MZI structure shows improved nonlinear dynamics and faster carrier recovery, making it suitable for scalable, high-speed all-optical processing. This study extends earlier research on CR-SOA-based logic gates and offers a multifunctional architecture that supports broader optical computing applications.

## Figures and Tables

**Table 1:** Default numerical parameters [11,12,15,16,17,18,19,20,21,22,23,24,25,26].

Symbol	Definition	Value	Unit
E_0_	Pulse energy	0.7	fJ
τ_FWHM_	Pulse width	1	ps
T	Bit period	8.33	ps
N	PRBS length	127	-
λ_A_	Wavelength of signal A	1550.7	nm
λ_B_	Wavelength of signal B	1549.3	nm
λ_CW_	Wavelength of signal CW	1557.3	nm
I	Injection current	250	mA
P_sat_	Saturation power	30	mW
τ_c_	Carrier lifetime	200	ps
τ_t_	Transition lifetime from CR to AR	5	ps
η	Population inversion factor	0.3	-
α	α-factor	5	-
α_CH_	Linewidth enhancement factor due to CH	1	-
α_SHB_	Linewidth enhancement factor due to SHB	0	-
ε_CH_	Nonlinear gain suppression factor due to CH	0.2	W^−1^
ε_SHB_	Nonlinear gain suppression factor due to SHB	0.2	W^−1^
τ_CH_	Temperature relaxation rate	0.3	ps
τ_SHB_	Carrier–carrier scattering rate	0.1	ps
Γ	Optical confinement factor	0.3	-
α	Differential gain	10^−20^	m^2^
N_tr_	Transparency carrier density	10^24^	m^−3^
L	Length of AR	500	μm
d	Thickness of AR	0.3	μm
w	Width of AR	3	μm
G_0_	Unsaturated power gain	30	dB
ω_0_	Central optical frequency	193.55	THz
N_SP_	Spontaneous emission factor	2	-
B_0_	Optical bandwidth	2	nm

**Table 2:** Truth table for both the encoder and comparator.

Input Signals		Output Encoder				Output Comparator		
A	B	$\bar{A}B$	$A\bar{B}$	AB (AND)	$\bar{A}\;\bar{B}$ (NOR)	$\bar{A}B$	$A\bar{B}$	$\mathrm{AB}+\bar{A}\;\bar{B}$ (XNOR)
0	0	0	0	0	1	0	0	1
0	1	1	0	0	0	1	0	0
1	0	0	1	0	0	0	1	0
1	1	0	0	1	0	0	0	1

**Table 3:** Performance comparison of encoder/comparator designs using different SOA technologies.

Ref.	Operation	Scheme	Speed (Gb/s)	Methodology	ER (dB)	QF	Performance Context
[22]	Comparator	QD-SOA	160	Simulation	10	9.00	Speed/QF benchmark
[23]	Comparator	SOA	10	Simulation	9	NR	Lower-speed baseline
[24]	Encoder	SOA	40	Experimental	10	NR	Experimental encoder reference
[25]	Encoder and comparator	SOA	40	Experimental	10	NR	Hybrid design reference
[26]	Comparator	SOA	40	Experimental	10	NR	Experimental comparator
This work	Encoder and comparator	CR-SOA	120	Simulation	10	8.33–9.05	Advanced speed/QF balance

(NR = Not Reported).

## Data Availability

Data are contained within the article.
